# Primary mitochondrial disease as a rare cause of unclear breathlessness and distinctive performance degradation – a case report

**DOI:** 10.1186/s12890-023-02391-x

**Published:** 2023-03-29

**Authors:** Ralf Ewert, Mohamed A. Elhadad, Dirk Habedank, Alexander Heine, Beate Stubbe

**Affiliations:** 1 Department of Internal Medicine B, Pneumology, University Hospital Greifswald, F.-Sauerbruchstr, 17475 Greifswald, Germany; 2 DRK-Hospital Berlin, Berlin, Germany

**Keywords:** Case report, Dyspnea, Invasive cardiopulmonary exercise testing, Lactate, Metabolic myopathy, Mitochondriopathy

## Abstract

**Background:**

Primary muscular disorders (metabolic myopathies, including mitochondrial disorders) are a rare cause of dyspnea. We report a case of dyspnea caused by a mitochondrial disorder with a pattern of clinical findings that can be classified in the known pathologies of mitochondrial deletion syndrome.

**Case presentation:**

The patient presented to us at 29 years of age, having had tachycardia, dyspnea, and functional impairment since childhood. She had been diagnosed with bronchial asthma and mild left ventricular hypertrophy and treated accordingly, but her symptoms had worsened. After more than 20 years of progressive physical and social limitations was a mitochondrial disease suspected in the exercise testing. We performed cardiopulmonary exercise testing (CPET) with right heart catheterization showed typical signs of mitochondrial myopathy. Genetic testing confirmed the presence of a ~ 13 kb deletion in mitochondrial DNA from the muscle. The patient was treated with dietary supplements for 1 year. In the course of time, the patient gave birth to a healthy child, which is developing normally.

**Conclusion:**

CPET and lung function data over 5 years demonstrated stable disease. We conclude that CPET and lung function analysis should be used consistently to evaluate the cause of dyspnea and for long-term observation.

## Background

Dyspnea can be caused by cardiac, pulmonary, and/or muscular disorders [1]. Muscular disorders in the context of cardiac or pulmonary disease are usually a secondary phenomenon; primary muscular disorders (metabolic myopathies, comprising disorders of glycogenolysis and glycolysis, mitochondrial function, and lipid metabolism [2]) are less common. Metabolic myopathy subtypes are associated with different findings in cardiopulmonary exercise testing (CPET) [3]. In particular, patients with mitochondrial disorders show an early and continuous increase in lactate under stress, leading to exaggerated circulatory and ventilatory responses [4]. Typical but not specific to any pattern is reduced anaerobic capacity (low VO_2_ @AT so-called ventilatory threshold VT_1_ and VO_2_peak) and increased ventilatory equivalents for O_2_ (VE/VO_2_) and CO_2_ (VE/VCO_2_).

If one considers primarily the muscular disorders as the cause of unclear shortness of breath and reduced performance, it becomes clear, that such changes in cardiac or pulmonary diseases often represent a secondary phenomenon. The clinical spectrum ranges from severe multisystem disorders in early childhood to isolated muscle spasms in adults. It is therefore easy to understand that the CPET parameters also show different patterns [6], whereby the “mitochondriopathies” are particularly characterized by an early and continuous increase in lactate under stress. In summary, patients are unable to adequately utilize oxygen for oxidative phosphorylation; and as consequence lactic acid accumulates early in exercise, which leads to exaggerated circulatory and ventilatory responses.

We report a case of dyspnea caused by a mitochondrial disorder (detected by CPET and confirmed by genetic testing) with a pattern of clinical findings that cannot be classified in the known pathologies of mitochondrial deletion syndrome [2, 5, 6].

## Case presentation

A 29-year-old woman was referred to our department for evaluation of unclear exertional dyspnea with extreme functional limitation.

She had experienced breathing difficulties and performance limitations since early childhood. A medical examination at 6 years of age had shown tachycardia, low blood pressure, and a normal electrocardiogram. Subsequently, her symptoms had worsened and she had developed severe pain and increasing weakness in her leg muscles. At 15 years of age, she was diagnosed with exercise-induced asthma. At 16 years of age, she underwent low-stress ergometry, which caused a sharp increase in heart rate there (a sharp increase in already at 20 watts from 100 to 160/min and at 60 watts to 200/min) with extreme shortness of breath. In CPET (bicycle, 3 min at 25 watts followed by 3 min at 50 watts), the respiratory quotient at rest was 1.02 and oxygen uptake (VO_2_) increased from 1.76 ml/min/kg to a maximum of 13.8 ml/kg/min. Echocardiography showed mild to moderate left ventricular hypertrophy, which declined after 3 years of beta-blocker therapy. She was not under beta-blocker therapy when referred to our department.

The patient was admitted to our department with pathological findings in ergometry (ramp protocol, increase of 15 watts/min up to 80 watts, reduced peak oxygen consumption with 8.5 ml/kg/min (29% predicted) with severe lactic acidosis (pH: 7.17; lactate: 25.8 mmol/L) and leading symptom of exercise dyspnea and inability to move her legs after physical exertion (walking 500 m). The clinical examination, laboratory analysis, and pulmonary function test showed no abnormalities. Inspiratory muscle strength was clearly impaired (maximal inspiratory pressure: 5.83 kPa [53% of normal], P0.1 0.10 (normal value 0.19), P0.1/Pi max 0.02 (normal value 0.02)). In CPET (3 min rest, 1 min unloaded cycling, then 16 watts/min intensification), a maximum load of 84 watts was achieved (60% of normal). Peak VO_2_ was 10.1 mL/kg/min (29% predicted), while the minute ventilation VE/VO_2_ ratio increased from 31 at rest to a maximum of 95 (Table 1). The leading reason for exercise termination was dyspnea followed by muscle weakness, sometimes dizziness and thoracic tightness without ECG-changes.

**Table 1:** Description of parameters in lung function analysis and exercise testing

parameter	time point							
	Aug 2017		Sep 2018		Aug 2020		Aug 2022	
**pulmonary function (unit)**	value	%predicted	value	%predicted	value	%predicted	value	%predicted
vital capacity VC (l)	3.9	97.4	4.1	103.2	4.1	104.8	4.0	94.0
Forced vital capacity FVC (l)	3.9	99.3	4.0	101.2	4.2	108.2	4.0	94.0
FEV1 (l)	3.4	98.4	3.5	103.0	3.5	104.0	3.5	98.0
FEV1 / FVC (%)	86.4		88.7		83.8		87.2	
total lung capacity TLC (l)	5.7	102.8	6.0	109.2	6.0	108.4	5.8	106.0
inspiratory capacity (l)	2.6	95.1	2.9	108.3	2.8	103.4	2.4	90.0
residual volume (l)	1.7	111.7	1.9	120.9	1.8	110.6	1.9	115.0
RV / TLC (%)	30.8	106.9	31.4	108.9	29.6	100.2	32.0	106.0
DLCO_c_ (mmol/min/kPa)	8.2	83.9	8.4	85.9	8.2	84.7	7.2	74.0
DLCO_c_ / VA (mmol/min/kPa/l)	1.6	91.2	1.7	92.8	1.6	88.2	1.4	77.0
**cardiopulmonary exercise testing**								
body weight (Kg)	65		66		66		56	
maximum power (Watt)	84	60	68	49	84	62	84	65
VO_2_@VT_1_ (ml/min)	507		443		312		397	
VO_2_@VT_1_ / VO_2_ peak pred. (%)	25.6		22.8		16.2		22	
peakVO_2_ (ml/min)	655	29	537	26	736	38	560	31
peakVO_2_ / weight (ml/min/kg)	10.1	29	8.5	30.8	11.1	29.2	10	31
peakVO_2_ / HR (ml)	3.6	29	4.4	28	4.1	40	4.3	27
maximum ventilation (l/min)	76		68		73		57	
maximum Vt_ex_ (l)	1.73		1.51		1.59		1.46	
breathing reserve (%)	45		53		49		60	
VE/VO_2_@rest	30.9		29.3		36.1		24.5	
VE/VO_2_@VT_1_	30.8		33.7		31		37.8	
VE/VO_2_@max	95		75		90		n.a.	
p_et_O2 @rest (mmHg)	115.26		122.67		116.56		111.13	
p_et_O2 @VT_1_ (mmHg)	116.24		120.87		113.33		121.86	
p_et_O2 @max (mmHg)	140.29		140.55		137.26		138.1	
VE/VCO_2_@rest	35.5		39.7		41.3		34.3	
VE/VCO_2_@VT_1_	35.7		40.5		38.2		33.9	
VE/VCO_2_@max	65.8		73.2		60.8		59	
p_et_CO_2_ @rest (mmHg)	30.28		21.74		30		30.77	
p_et_CO_2_ @VT_1_ (mmHg)	29.65		25.47		30.69		30.73	
p_et_CO_2_ @max (mmHg)	14.9		22.68		n.a.		19.01	
maximum RER	1.96		1.86		1.59		1.93	
**blood gas analysis (at rest)**								
AaDO_2_ (mmHg)	36.14		11.47		27.1		29.32	
p_a−et_CO_2_ (mmHg)	0.66		4.34		-1.32		-1.12	
p_a_O_2_ (mmHg)	77.1		99.6		85		82	
p_a_CO_2_ (mmHg)	31.3		28.8		29		31.6	
S_a_O_2_ (%)	96.05		97.88		96.96		95.9	
lactate (mmol/l)	2.4		1.9		1		1.6	
HCO_3_^−^ (mmol/l)	22.3		21.8		21.9		23	
base excess (mmol/l)	-6.3		-4.6		-4.6		-4	
VO_2_ (ml/min)	377		329		215		216	
**blood gas analysis (at 46 W)**								
AaDO_2_ (mmHg)	15.66		15.46		18.01		10.56	
p_a−et_CO_2_ (mmHg)	3.29		3.61		4.31		-1.12	
p_a_O_2_ (mmHg)	113		116		109		119	
p_a_CO_2_ (mmHg)	30.4		25.9		28.1		29.3	
S_a_O_2_ (%)	98.9		98.5		98.5		99.1	
lactate (mmol/l)	6.2		4.1		2.3		5.4	
HCO_3_^−^ (mmol/l)	19.4		20.7		20.7		19.2	
base excess (mmol/l)	-6.3		-4.6		-4.6		-4	
VO_2_ (ml/min)	519		433		544		425	
**blood gs analysis (at 68 W)**								
AaDO_2_ (mmHg)	15.98		17.72		17.46		n.a.	
p_a−et_CO_2_ (mmHg)	3.82		6.71		4.66		5.13	
p_a_O_2_ (mmHg)	120		117		112		135	
p_a_CO_2_ (mmHg)	21.6		22.6		24.8		25.7	
S_a_O_2_ (%)	98.9		98.5		98.3		99.1	
lactate (mmol/l)	14.4		7.5		6.3		10.8	
HCO_3_^−^ (mmol/l)	14.8		18.7		18.1		14.7	
base excess (mmol/l)	-13		-7.2		-8		-8.7	
VO_2_ (ml/min)	570		478		640		448	

Abbreviations: AaDO_2_ arterial-alveolar difference for oxygen, VT_1_ ventilatory threshold, DLCO_c_ diffusion capacity of carbon monoxide corrected by haemoglobin, DLCO_c_ / VA Krogh factor, FEV1 forced expiratory volume in one second, FVC forced vital capacity, HCO_3_^−^ bicarbonate ion, HR heart rate, peakVO_2_ peak oxygen uptake, p_a_CO_2_ arterial partial pressure of carbon dioxide, p_a_O_2_ arterial partial pressure of oxygen, p_a−et_CO_2_ arterial - end-tidal difference for carbon dioxide, p_et_CO_2_ end- tidal pressure of carbon dioxide, p_et_CO_2_@VT_1_ end tidal pressure of carbon dioxide at ventilatory threshold, RER respiratory ratio (quotient of carbon dioxide output and oxygen uptake), S_a_O_2_ arterial oxygen saturation, TLC total lung capacity, VE/VCO_2_@VT_1_ ratio ventilation to carbon dioxide output at ventilatory threshold, VE/VCO_2_@max ratio ventilation to carbon dioxide output at maximum exercise, VE/VCO_2_@rest ratio ventilation to carbon dioxide output at rest, VE/VO_2_@VT_1_ ratio ventilation to oxygen uptake at ventilatory threshold, VE/VO_2_@max ratio ventilation to oxygen uptake at maximum exercise, VE/VO_2_@rest ratio ventilation to oxygen uptake at rest, VO_2_ oxygen uptake per minute, VO_2_@VT_1_ oxygen uptake at ventilatory threshold, VO_2_/HR oxygen uptake per heart rate, Vt_ex_ expiratory tidal volume

Echocardiography showed mild hypertrophic left cardiomyopathy, without signs of pulmonary hypertension.

**Fig. 1:**
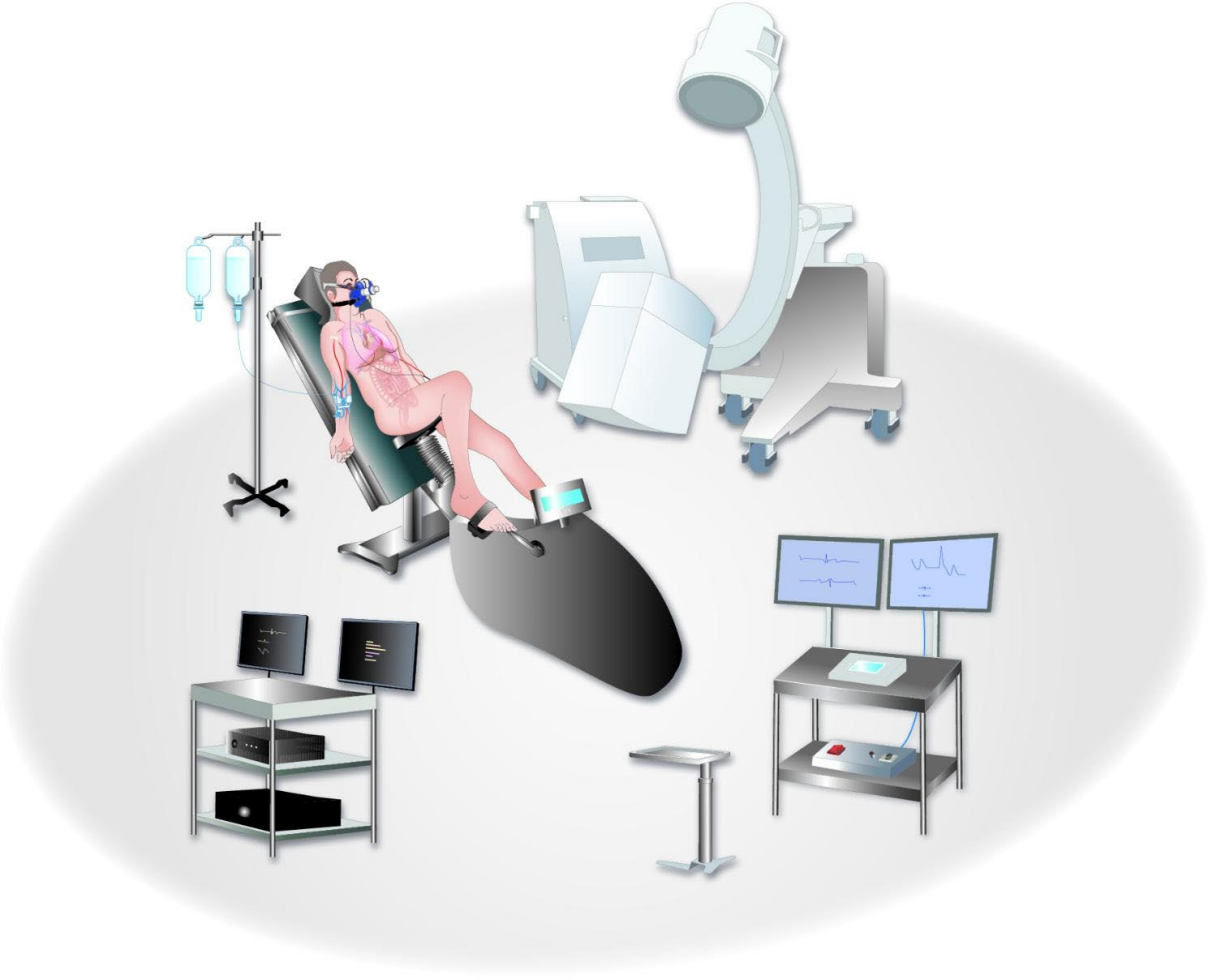
Invasive cardiopulmonary exercise testing combined with right heart catheterization in semi-supine position.

**Fig. 2:**
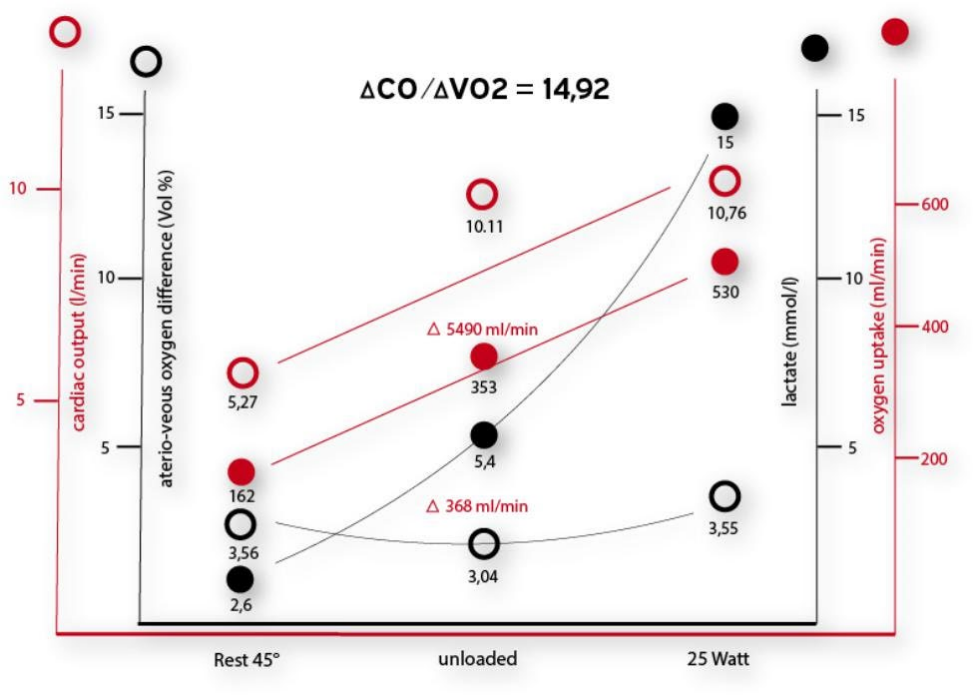
Cardiopulmonary exercise testing combined with right heart catheterization shows a rapid increase in lactate level, hyperdynamic circulatory adaptation, and low arteriovenous oxygen difference, consistent with the presence of a mitochondrial disorder. Δ = change; CO = cardiac output; VO_2_ = oxygen uptake

Invasive CPET (combined with right heart catheterization in semi-supine position, Fig. 1) showed a normal pulmonary pressure at rest (PAPmean 15 mmHg) and at 25 W (PAPmean 24 mmHg and with normal pulmonary vascular resistance (PVR < 1,5 WU at all measurements, Table 2). The mixed venous oxygen saturation was constant over the whole exercise time. The lactate concentration was 15.0 mmol/L at 25 watts (Fig. 2) and fell to 7.0 mmol/L at rest over 15 min.

**Table 2:** Invasive cardiopulmonary exercise testing

parameter	unit	baseline	45° upright position	unloaded cycling	exercise 25 W
heart rate	min^− 1^	80.0	81.0	120.0	157.0
systolic blood pressure	mmHg	115.0	126.0	134.0	135.0
diastolic blood pressure	mmHg	72.0	79.0	89.0	77.0
mean systemic arterial pressure	mmHg	99.0	90.0	113.0	99.0
PAWP	mmHg	7.0	9.0	9.0	15.0
CVP	mmHg	7.0	6.0	7.0	13.0
VO_2_ -table	l/min	193.7			
VO_2_ -measured	l/min	223.0	162.0	353.0	530.0
CO (iFM)	l/min	5.8			
CO (dFM)	l/min	6.7	4.5	11.6	14.9
SVR (iFM)	WU	15.9			
PVR (iFM)	WU	1.4			
CI (iFM)	l/min/m²	3.3			
SVR (dFM)	WU	13.8	18.5	9.1	5.8
PVR (dFM)	WU	1.2	1.3	0.7	0.6
CI (dFM)	l/min/m²	3.8	2.6	6.6	8.5
CO (TD, mean)	l/min	6.3	5.3	10.1	10.8
SVR (TD, mean)	WU	14.7	15.9	10.5	8.0
PVR (TD, mean)	WU	1.3	1.1	0.8	0.8
CI (TD, mean)	l/min/m2	3.6	3.0	5.7	6.1
PAP_sys_	mmHg	20.0	18.0	30.0	35.0
PAP_dia_	mmHg	8.0	8.0	8.0	14.0
PAP_mean_	mmHg	15.0	15.0	17.0	24.0
p_a_O_2_	mmHg	98.8	103.0	106.0	126.0
p_a_CO_2_	mmHg	29.9	29.6	28.4	21.2
S_a_O_2_	%	97.5	97.6	98.2	98.0
S_v_O_2_	%	79.5	78.4	81.8	78.9
t_a_O_2_	Vol%	18.1	18.1	18.2	18.2
t_v_O_2_	Vol%	14.8	14.6	15.2	14.6
a-vDO_2_	Vol%	3.3	3.6	3.0	3.5
lactate, arterial	mmol/l	3.3	2.6	5.4	15.0

Abbbreviations: a-vDO_2_ arterial-venous volume difference for oxygen, CI cardiac index, CO cardiac output, CVP central venous pressure, dFM direct method of Fick, iFM indirect method of Fick, p_a_CO_2_ arterial partial pressure of carbon dioxide, p_a_O_2_ arterial partial pressure of oxygen, PAP_dia_ diastolic pulmonary artery pressure, PAP_mean_ mean pulmonary artery pressure, PAP_sys_ systolic pulmonary artery pressure, PAWP pulmonary arterial wedge pressure, PVR pulmonary vascular resistance, S_a_O_2_ arterial oxygen saturation, S_v_O_2_ venous oxygen saturation, SVR systemic vascular resistance, t_a_O_2_ arterial oxygen tension in percent, t_v_O_2_ venous oxygen tension in percent, TD thermodilution, VO_2_ oxygen uptake per minute, WU wood unit

Cardiac output, cardiac index and resistances were calculated by thermodilution (TD) and direct and indirect method of Fick (dFM resp. iFM), with the iFM method using the table of LaFarge.

Histological analysis of a muscle biopsy showed no abnormalities or mitochondrial disorders (no ragged red fibers in Gomori trichrome stain). Histochemical analysis showed significantly increased citrate synthase activity, reduced activity of complex I and IV, and normal activity of complex II and III. Mitochondrial DNA from the muscle showed a deletion of bases 3,261 to 16,068 with 36% heteroplasmy.

After the diagnosis, the patient, at her own request, received dietary supplements (including coenzyme Q10/ubiquinol, vitamin B1/B6 complex, alpha-lipoic acid, L-taurine, magnesium, and potassium) for a year.

The patient’s symptoms persisted and lung function and CPET data remained unchanged over 5 years (Table 1). After thorough genetic counseling, the patient had an uncomplicated pregnancy resulting in the birth of a healthy boy (c-Sect. 05/2019, male, 55 cm, 4150 g). who is developing normally.

## Discussion

In this case of dyspnea, the suspicion of mitochondrial disease was raised only after ~ 20 years of progressively worsening functional limitation. Although the clinical pattern of mild hypertrophic left cardiomyopathy and muscle weakness did not match the known pathologies of mitochondrial deletion syndrome [2, 5, 6], CPET findings were consistent with mitochondrial myopathy. Small case series [7–9] have previously shown significantly reduced maximum VO_2_ and increased VE/VO_2_ in patients with mitochondrial disorders compared with control individuals. Hyperdynamic circulatory adaptation and reduced arteriovenous oxygen difference were previously identified in a study of 40 patients with mitochondrial myopathy compared with healthy sedentary individuals [7] and allow differentiation of mitochondrial myopathy from muscular deconditioning due to cardiac and pulmonary diseases [10]. Further, as shown in this case elevated lactate concentrations in blood may be a clue to impaired aerobic energy metabolism [11].

Treatment options for primary mitochondrial disease are limited and may include physiotherapy and dietary supplements [5, 6, 12]. Although the latter did not produce any clinical improvement in our patient, such therapy should be evaluated cautiously. We clearly emphasize that exercise therapy may be a therapeutic solution in these patients, in particular, aerobic endurance training can increase mitochondrial mass, by stimulating mitochondrial biogenesis, and increase muscle mitochondrial enzyme activities and muscle strength [12, 13].

Although the pregnancy and subsequent development of the child were uneventful in our case, it should be noted that the offspring of women with mitochondrial DNA deletion disorders have a ~ 4% risk of inheriting the deletion [14]. Patients with mitochondrial diseases have been reported to have worsening of symptoms and an increased rate of complications during pregnancy, and their children tend to have more congenital anomalies than expected [14]. Our patient had no complications during pregnancy and delivery. The now almost four-year-old child also shows no abnormalities. In a retrospective analysis of pregnancies of patients with mitochondrial diseases (38% with myopathies) worsening of symptoms and findings during pregnancy and an increased rate of complications (gestational diabetes, preeclampsia) were found. The newborns were born on their expectation date but tended to have more congenital anomalies than expected [15].

## Conclusion

In summary, this case demonstrates the importance of CPET in evaluating unclear dyspnea, both for diagnosis and long-term monitoring.

## Data Availability

The data can be made available by contacting the corresponding author.

## Abbreviations

CPET: Cardiopulmonary exercise testing
VE: Minute ventilation
VO_2_: Oxygen uptake
